# Stimulants

**Published:** 1861-04

**Authors:** 


**STIMULANTS.**—Close observation and correct physiological research reach the same conclusion, that hearty eating and steady, hard work in the open air give the highest degree of bodily vigor, endurance, and lastingness. Such persons have a strong appetite and a rapid digestion, which speedily converts the food into nutriment, and the labor as rapidly works the old and useless particles out of the system; hence, the newer a man is, the harder he works and the heartier he eats. But suppose, in addition, he drinks liquor largely. Its effect is to arrest the metamorphosis of the tissues, to keep longer in the body what ought to have been worked off; hence he soon becomes over-full; his skin becomes distended, and he is always "tight!" (a very expressive phrase, that.) It is clear, then, that whatever arrests waste, arrests the change which ought to take place in corporeal particles, preparatory to their being conveyed away out of the system, and is unnatural and pernicious.

A man who has a bottle of rum will survive his friend at sea or in a desert, simply because it is the nature of alcohol to arrest waste and decay, up to a certain point.

So it is with a man who studies hard, who works the brain. Alcohol has an affinity for the brain. Within an hour after a glass of brandy is swallowed, more of it is found in a given quantity of brain than in any equal quantity of blood. This was demonstrated twenty years ago by Dr. Percy, so we rest here with a bare statement of the fact. Hence, a man with a glass of toddy will think longer, his brain will longer work with activity, than if he had none, up to a certain point, because it arrests the metamorphosis of the tissues of the brain. Coffee and tea do the same thing, and so does tobacco. Thus it was that during the Irish famine, a dozen years ago, it was often made a subject of remark, that when an almost starving wretch chanced to get a little money, it was expended in tea or tobacco or spirits; and when asked the reason, the same reply was made, "It went farther" than any thing else; it was concentrated carbon, no bone, no husk, not an atom of waste.

We saw the greatest intellect of this nation, on being called to make a speech in the old Jenny Lind Hall on Broadway, so drunk that he could scarcely articulate a distinct word; the syllables ran into each other in sound; and he had to hold himself up by the corner of the table which opportunely stood by.

It is reported of the greatest divine that ever trod on this continent, that he suddenly stopped in a sermon, raised his hand to his brow, and exclaiming, "God, as with a sponge, has blotted out my mind," burst into tears, and sat down. He had fallen into habits of drinking spirits.

One of the best writers on doctrine in a most influential denomination, and some of whose productions are made standards in his church, was a drunken man, and died of the chronic diarrhea, which often ends a drunkard's life.

These men were at times compelled to brain-work when it was not in working order, when it was tired, exhausted; but some engagement, some inexorable necessity, as they thought, demanded the effort, and they found out in one way or another that a cup of tea or a glass of toddy enabled them to come up to the task, and thus the habit grew on them, until at last they found themselves under the necessity of "taking a drink" before making a public address, and sometimes before its preparation. But tea and alcohol are transient in their effects. Suppose a man has to be one of several speakers, and happens to miscalculate on the length of the addresses which precede his, the inevitable result is, a disgraceful failure or a new potation; but suppose he couldn't get it!!

So that a cup of strong tea will enable a man to get more work out of his brain than would otherwise have been done. But this is expenditure before an income; and for a while the evil day of bankruptcy of brain may be deferred, but its eventual coming is inevitable, and with it the ruin of the mind and of the man. The conclusion of the whole matter is, that the man who drinks a cup of tea or a glass of brandy to enable him the better to discharge any public service, is already on the high-road to dishonor and a drunkard's death. Young clergymen, politicians, lawyers, doctors, beware!! Greater than any of you can ever hope to be, have made shipwreck of soul, body, and estate on this very rock, and why may not you? The reason of this infinitely inevitable result is, that stimulants arrest waste—this is their inherent, peculiar, distinctive, specific quality; this habitual arrest clogs up, and sooner or later the wheels of life stand still forever.

